# Examining the Impact of COVID-19 on Education and Service Access for Diverse Families of Young Children With and Without Developmental Delays

**DOI:** 10.1177/10664807231163261

**Published:** 2023-03-14

**Authors:** Ann Marie Martin, Laura Lee McIntyre, Cameron Neece

**Affiliations:** 1 Indiana University, School of Medicine, Indianapolis, IN, USA; 2 3265 University of Oregon, College of Education, Eugene, OR, USA; 3 Department of Psychology, 4608 Loma Linda University, Loma Linda, CA, USA

**Keywords:** developmental disability, education, parenting stress, COVID19, latinx

## Abstract

The rapid transition to virtual learning due to the COVID-19 pandemic created unprecedented challenges that significantly impacted caregivers of young children, particularly those with developmental delays and children from non-English speaking households (Valicenti-McDermott et al., 2022). The current study aims to describe caregivers’ concerns regarding the COVID-19 pandemic in general and specific educational concerns following school closures, and to determine whether their concerns varied as a function of whether the child had a developmental delay or the parent's ethnicity. Results revealed that caregivers of children with DD endorsed a greater number of general and education-specific COVID-19 concerns compared to caregivers of TD children, and non-Latinx caregivers of children with DD reported more general COVID-19 concerns compared to Latinx caregivers of children with DD. With respect to education-specific concerns, caregivers of children with DD reported a greater impact from the loss and/or delay of services and reported feeling significantly less capable of conducting educational activities in the home compared to caregivers of TD children. However, almost all caregivers in the study endorsed some level of stress from remote instruction. These findings suggest there is a specific need for attention to caregiver mental health and an examination of long-term educational outcomes resulting from extended school closures during the COVID-19 pandemic.

The global COVID-19 pandemic and concurrent stay-at-home order in California halted in-person educational activities and related services for most children. As a result, these activities were delivered virtually in the early period of the pandemic (Easop, 2022; Viner et al., 2020) and most caregivers were asked to step in and facilitate academic classes and educational services for their children. As the pandemic continued, schools continued to rely on distance learning strategies to maintain social distancing policies, despite a number of challenges faced by educators, children, and caregivers (Supratiwi et al., 2021). Additionally, caregivers of children with developmental delays (DD) were asked to implement developmental services (e.g., occupational therapy, adapted physical education) in the home setting (Allison & Levac, 2022).

A burgeoning body of literature has begun to examine the impact of school closures on caregivers’ experiences and results have been mixed. Most notably, there was variability in each household's capacity to support children's educational activities and provide adequate resources (e.g., time, experience, technology), widening the educational inequalities that already existed in the system (Doyle, 2020; Supratiwi et al., 2021). Families from lower socioeconomic backgrounds typically experienced greater disadvantages, including difficulties with unreliable internet and lack of appropriate electronic devices (National Center for Education Statistic, 2021). Children with DD required additional support to engage in the educational environment, and parents of children with significant learning needs were unlikely to be equipped with the resources necessary to conduct effective remote learning (Masonbrink & Hurley, 2020; Valicenti-McDermott et al., 2022). However, some families reported positive outcomes from the school closures, such as spending more time together (Latzer et al., 2021; Neece et al., 2020), educational flexibility (Lipkin & Crepeau-Hobson, 2022), and special education teachers endorsed the potential for virtual learning to increase access to education for children with complex needs through improved teacher and parent training and higher quality learning platforms (Karasel Ayda et al., 2020).

## Educational Losses for Children with Developmental Delays

Individuals with DD may receive several educational services and often rely on in-person support for personal care, social interaction, and educational activities, resulting in a significant impact on access to specialized care when services were halted indefinitely at the start of the pandemic (Constantino & Sahin, 2020; Valicenti-McDermott et al., 2022). In addition, children with DD often rely on schools for a variety of necessary supports beyond traditional academic instruction, including communication and social skill programs (Stenhoff et al., 2020). A report on education equity during COVID-19 revealed that many school districts across the United States significantly reduced or eliminated all services for students with disabilities (Easop, 2022). This is especially impactful in rural and geographic regions with lower SES, where schools may be the only providers of specialized services and evidenced-based programs for children with DD. These services may be difficult to replicate in a virtual format due to difficulties in maintaining the child's attention and self-regulation, digital literacy, and technological limitations (Stenhoff et al., 2020). Moreover, research suggests that the transition to remote learning had a greater effect on children receiving special education (as compared to children in general education) due to the difficulties in recreating the learning environment specified in the child's individualized education plan (IEP), a core facet of the Individuals with Disabilities Education Act (IDEA), the law that regulates education for students with disabilities (Easop, 2022; IDEA, 2004; Karasel Ayda et al., 2020; Petretto et al., 2020).

Children with special needs often rely on redirecting, prompting, and guidance during educational activities, and when school and services were halted, the responsibility was shifted primarily to caregivers to fulfill the role of the teacher, educational aide, and therapist, and pressured families to replicate the classroom environment in an effort to reduce potential regression in skills (Constantino & Sahin, 2020). Once services and educational activities were transitioned to virtual formats, disparities were highlighted underscoring a general lack of accessible tools and resources for students with disabilities to navigate and engage in online learning (Tlili et al., 2021). This was especially relevant for young children with DD who rely on reasonable accommodations for equal opportunity to engage in educational activities and may require nontraditional formats of demonstrating their understanding of the academic content, all functions that may be difficult to replicate in a virtual format (Karasel Ayda et al., 2020; Petretto et al., 2020; Stenhoff et al., 2020). For example, when conducting interviews regarding virtual learning for children with special needs, teachers endorsed difficulties with creating interactive educational programs that provide feedback immediately, a lack of tailored materials and manipulatives that support lessons, and reduced access to specialists who can aid in the modification of educational activities (Karasel Ayda et al., 2020).

Research on the impact of virtual learning on children is emerging, but some studies have estimated that the school closures could result in up to 12-months of learning loss (Easop, 2022). The migration to distance learning was rapid and unplanned, thus virtual learning strategies were likely underdeveloped making it especially challenging for special education teachers to attend to children of different needs through a computer screen, again placing the emphasis on caregivers to supplement professional support (Petretto et al., 2020). However, a few studies have found that some students thrived in the absence of bullying and the social demands of in-person learning (Lipkin & Crepeau-Hobson, 2022; Reicher, 2020). Additionally, there is a potential for vast improvement in online learning which could increase caregivers’ access to individualized coaching to support their child's educational activities at home (Steed et al., 2022).

## The Impact on Parents and Caregivers

While the impact of school closures and increased demands for parent involvement in educational activities is still emerging, research to date has produced mixed results. Research from Shaw and Shaw (2021) identified several themes from a qualitative questionnaire with 137 parents of TD and DD school-aged children (i.e., 4–18 years old) from the United Kingdom who had taken a primary role in their child's educational activities. The themes include a perceived lack of ability to educate their child effectively at home, a change in family dynamics as a result of the dual role (caregiver & education professional substitute), and the need for a routine in order to address emotional needs (Shaw & Shaw, 2021). Research has also indicated that despite agreeing with in-person school closures, parents struggled with motivating their child to engage in educational activities and the production of satisfactory learning outcomes and socio-emotional development (Garbe et al., 2020). Caregivers of children with DD faced additional hardships, such as an increase in maladaptive behaviors and skill regressions that added to the emotional toll that caregivers were already experiencing (Latzer et al., 2021). Additionally, parents of children with DD reported higher levels of parenting stress, depressive symptoms, and anxiety symptoms as compared to parents of typical-developing (TD) children during this time (Chan & Fung, 2022; Sideropoulos et al., 2022), highlighting the vulnerability of this population.

While caregivers of children with DD tend to report higher levels of parenting stress compared to caregivers of TD children in general, those with higher levels of parenting stress pre-pandemic were also more likely to report an increase in the intensity of their anxiety (i.e., state anxiety) during the pandemic (Ren et al., 2020) and self-report symptoms of depression and psychological distress (Chan & Fung, 2022). Despite the additional hardships, research has found that parents of children with DD with greater support systems and better coping skills demonstrated greater resiliency (Latzer et al., 2021). Some of the most effective coping skills reported by parents in Neece et al. (2020) included behavioral strategies (e.g., reinforcement systems), routines, family activities (e.g., walks), and meditation techniques. Additionally, some families endorsed positive experiences from the lockdown such as the ability to spend more time with their children (as opposed to working outside the home for the majority of the day), strengthening of the family unit, and witnessing certain skills they were unaware their child was able to demonstrate (Latzer et al., 2021; Neece et al., 2020).

## Ethnic Differences

Although there is a relative lack of research on the effects of the pandemic on Latinx families, there are a few articles highlighting the impact of COVID-19 on this demographic. Most notably is the identification of the dual risk status of Latinx caregivers of young children with DD (Suarez-Balcazar et al., 2021). Historically a marginalized population, Latinx communities often have limited access to quality health care, are of lower socio-economic status, and may have less social support especially if they are immigrant families, all risk factors for high mental health concerns (Suarez-Balcazar et al., 2021). Latinx caregivers of youth with DD also faced additional challenges at home, with a loss of essential educational and developmental services, placing a burden on underprepared caregivers to support their child's complex needs, both economically and socially, resulting in a profound effect on the family unit (Neece et al., 2020; Suarez-Balcazar et al., 2021). The return to in-person school and services stands as a key determining factor in long-term consequences of the pandemic on Latinx communities; however, reopening decisions have favored more affluent areas with White students being more likely to attend school in-person when schools first reopened in the 2020–2021 school year (Camp & Zamarro, 2021; Kogan, 2021), resulting in a longer duration of virtual learning for Latinx and other minority communities, likely exacerbating educational inequalities (Fox et al., 2021). All of these factors combined highlight the need for a research focus on Latinx caregivers of children with DD in order to inform future interventions to support these families to mitigate their dual risk status and long-term consequences resulting from the loss of education and services from the COVID-19 pandemic.

## The Present Study

The COVID-19 school closures and shift to remote learning significantly impacted the educational activities of young children, especially young children with DD. Emerging research has identified widening gaps in educational inequalities and variability of resources available to families to shoulder the burden on remote learning (Doyle, 2020; Haderlein et al., 2021). In addition, school closures have had a considerable impact on appropriate education and service provision (Constantino & Sahin, 2020), parental mental health (Shaw & Shaw, 2021), and a profound effect on Latinx caregivers of children with DD (Suarez-Balcazar et al., 2021). Despite these initial findings, research examining the impact of remote learning on young children with and without DD is still emerging. Taking into consideration the gaps in the literature on the effects of the COVID-19 pandemic on Latinx families and the education of young children with DD, the current study seeks to address the following questions: (1a) What were caregivers’ general concerns regarding COVID-19? (2) What were caregivers’ educational concerns following school closures? (3) Did children with DD experience greater disruptions to education as compared to TD children? And, (3b) What role did ethnicity play in access to education and services for children with DD?

## Methods

### Participants

Study participants were 147 parents of children (aged 3–9 years, *M* = 5.05 SD = 1.19); 111 caregivers of children with developmental delays (DD) and 36 caregivers of typically developing (TD) children. The families of children with DD were part of a larger study examining the effectiveness of parent stress-reduction and behavioral parent training interventions for this population. Families of children with DD, were located in two states Oregon (*n* = 25) and California (*n* = 86). California participants were primarily recruited through the Inland Empire Regional Center, a government agency that provides services for all individuals with developmental disabilities in the Inland Empire. Participants in Oregon were recruited through a local school district near Portland, Oregon. For participants in the TD group (all located in California), families were recruited through local preschools, social media postings, the Inland Empire Regional Center, Head Start Program, and a local federally qualified health center.

Inclusion criteria for the DD group were: (a) having a child ages 3 to 5 years with a DD who was receiving early intervention or early childhood/preschool special education through an individualized family service plan (IFSP) or IEP; at time of entry into the intervention; (b) having parent-reported concerns about their child's behavior (reporting some or many concerns about the frequency or intensity of child challenging behavior at study entry), and (c) parent must speak English or Spanish. Parents were excluded from participation in the intervention if (a) they screened positive for active psychosis, substance abuse, or suicidality (endorsement of screening questions for associated modules of the Structured Clinical Interview for DSM Disorders (SCID)-Research Version Non-Patient Edition) (First et al., 2002); (b) they were currently receiving any form of psychological or behavioral treatment at the time of referral (e.g., counseling, parent training, parent support group); or (c) their child had significant sensory impairments (e.g., deafness, blindness) or nonambulatory conditions that would necessitate the need for significant modifications to the study protocol.

Inclusion criteria for the TD group of the intervention were having a child between 3 and 5 years of age and English as the primary language in the home. Exclusionary criteria for those in the TD group included: (a) community diagnosis of ASD or other developmental delay (e.g., Intellectual Disability, Global Developmental Delay); (b) having a sibling with ASD or other developmental delay; (c) a total score of 12 or higher on the Social Communication Questionnaire, which is the recommended cutoff used to determine whether a younger child is likely to have ASD (Corsello et al., 2007; Rutter et al., 2003); (d) Abbreviated Battery IQ score below 85 on the SB-5 (Roid et al., 2003); and (e) children with debilitating physical disabilities that prevent them from participating in the assessment tasks described in the procedures (e.g., child is not ambulatory).

The first two research questions compared families of children with DD to families of children with TD, and the third question utilized data from the DD sample only and compared Latinx to non-Latinx parents of children with DD. Sample demographics and group differences are summarized in Table 1. In the TD group, the children were significantly older and the participating parent was less likely to be female and was younger on average compared to parents in the DD group. Significant differences were also observed in the utilization of social services including SSI, Medicaid/MediCal, CalFresh/Food Stamps, and WIC. With regards to the within group differences within the DD sample by ethnicity, significant differences were observed in caregiver's highest level of education whereby a higher percentage of Latinx caregivers (36% of whom were monolingual Spanish-speaking) had a high school diploma or less as compared to non-Latinx caregivers. In addition, the Latinx sample was less likely to be employed outside the home, reported less income than non-Latinx participants, and had higher rates of accessing social service programs including SSI, Medicaid/MediCal, CalFresh/Food Stamps, and WIC. Two of the DD child characteristics resulted in significant differences: a diagnosis of ASD was more prevalent in the Latinx group and more Latinx children were enrolled in school. These variables were considered as covariates in the analyses.

**Table 1.** Demographics Characteristics.

Variable	TD sample (n = 36)	DD sample (n = 111)	TD/DD *X^2^*	DD Latinx (n = 61)	DD sample non-Latinx (n = 50)	DD Latinx/Non *X^2^*
Child Gender (% Male)	53	64	1.4	67.2	60	0.6
Child Mean Age (SD)	5.6 (1.1)	4.9 (1.2)	3.3**	4.9 (1.1)	4.9 (1.2)	−0.2
PC Gender (% Female)	86	98	8.8**	96.7	100	1.7
PC Mean Age (SD)	34.9 (6.2)	38.2 (8.1)	−2.2*	37.4 (8.4)	39.1 (7.6)	−1.1
PC Race			9.6^†^			—
% White	58.3	36	—	0	72	—
% Latino	44.4	55	—	100	0	—
% Black	2.8	9.9	—	0	20	—
% Other	5.6	7.2	—	0	16	—
PC Primary Language (% Spanish)	5.6	36	13.7***	65.6	0	43.5***
Marital Status (% Married)	80.6	70.3	3.7	67.2	74	7.2
Education (% with High School Diploma or less)	11.1	34.2	13.1	50.8	14	39.4***
Employment (% Employed Outside the Home)	47.2	44.5	9.0	35	56	15.5*
Income (% less than $30,000)	11	25.2	1.2	39.3	8	−5.7**
Social Services						
Supplemental Security Income (%)	0	19.8	8.3**	31.1	6	10.9**
Medicaid/Medical (%)	0	66.7	48.3***	80.3	50	11.4**
CalFresh/Food Stamps (%)	0	24.3	10.7**	32.8	14	5.3*
Women, Infants, Children (WIC; %)	0	25.2	11.2**	34.4	14	6.1**
Diagnosis (% ASD)	—	51.4	—	63.9	36	13.4*
Receiving Services for DD (%)	—	87.3	—	83.6	90	1.7
Is the Child Enrolled in Schol (% Yes)	89	85	.27	95	73	10.2**
Special Education Eligibility (% Enrolled)	—	66.7	—	76.7	56	3.9
How much has your child been impacted by a loss and/or delay of services on a scale 1–10 (M:SD)	4.63 (3.35)	6.81 (2.72)	—	7.10 (2.76)	6.46 (2.64)	—

†*p* < .1 *p < .05 **p < .01 ***p < .001.

### Procedures

Procedures were approved by the Institutional Review Board at Loma Linda University. Participants were contacted from November 2020 to February 2021 to complete an online battery of questionnaires assessing their experiences during the pandemic. Data was collected online via Qualtrics and analyses for the study were conducted using version 27 of SPSS.

### Measures

Measures for the current study were administered in the participant's native language. For Spanish measures, study personnel translated the questionnaires into Spanish and then back translated the Spanish forms to check for accuracy.

#### Demographic form

Caregivers completed a demographic form with information about parent and child characteristics (e.g., parent and child age, child diagnosis) and socioeconomic status (e.g., family income, employment, parent level of education).

#### COVID-19 questionnaire

The COVID-19 questionnaire was developed by our research team to examine the impact of COVID-19 on families in our research studies. The parent report measure contains 50 questions about different experiences related to the pandemic and current events across five domains: natural disasters, protests, COVID-19, family impact, and impact on education and services. Participants were asked to rate each item on a Likert scale or to choose from a list of options to select (e.g., *what are your concerns for your child over the next year or two due to the COVID-19 pandemic? Please mark all that apply*). The current paper focused on 12 questions in this measure assessing COVID concerns, educational concerns, and COVID's impact on child's education and services.

## Results

Descriptive analyses were calculated for the study. We performed a series of frequencies, chi-squares, and t-tests to determine significant differences in self-reported experiences between the two comparison groups (TD compared to DD and DD-Latinx compared to DD-non-Latinx). Demographic descriptive statistics between English and Spanish-speaking Latinx participants were inspected but no significant group differences were observed so the Latinx sample was utilized as a whole. We also considered possible covariates in demographic variables that were different between developmental status and ethnic status. If the variables were significantly related to the dependent variable, the covariates were controlled for in the analyses.

### COVID-19 Concerns

 Table 2 provides information on COVID-19 concerns by demographic group, chi-square and t-test statistics are included. Results for developmental status (DD compared to TD) revealed five significant group differences in concerns related to the pandemic. Parents of children with DD were significantly more concerned about the ramifications of loss of services (*X^2 ^*= 11.51, *p < *.001), child behavior problems stemming from anger (*X^2 ^*= 4.55, *p < *.05), and a loss of communication (*X^2 ^*= 6.01, *p < *.01), motor (*X^2 ^*= 7.28, *p < *.01), and academic skills (*X^2 ^*= 4.25, *p < *.05) related to the stay-at-home order. DD caregivers on average endorsed a significantly greater number of pandemic-related concerns compared to TD caregivers (*t^ ^*= −1.92, *p < *.05).

**Table 2.** Frequencies and Chi-square Results for COVID Concerns by Demographic Group.

Variable	DD	TD	X^2^	DD – Latinx	DD – Non-Latinx	X^2^
Catching COVID (% yes)	69	75	.55	75	60	3.02^†^
Other Physical Health Concern (% yes)	19	11	1.17	23	14	1.44
Loss of Services (% yes)	49	17	11.51***	46	52	.41
Anxiety (% yes)	60	47	1.66	57	62	.24
Depression (% yes)	37	39	.04	26	50	6.67**
Anger (% yes)	45	25	4.55*	44	46	.03
Loneliness (% yes)	52	64	1.49	41	66	6.89**
Communication Skills (% yes)	54	31	6.01**	48	62	2.31
Social Skills (% yes)	69	67	.04	57	82	7.72**
Motor Skills (% yes)	23	3	7.28**	20	26	.63
Academic Skills (%yes)	61	42	4.25*	57	66	.86
Screen Time (% yes)	53	58	.29	44	64	4.30*
Diet (% yes)	29	25	.20	23	36	2.28
Fighting with Siblings (% yes)	32	31	.01	33	30	.10
Total Count (M:SD)	6.5(3.25)	5.33 (2.84)	−1.92*	5.95 (3.1)	7.16 (3.33)	−1.98*

†*p* < .1 *p < .05 **p < .01 ***p < .001.

Within the DD group, analyses examining ethnic group differences (Latinx compared to non-Latinx) in the DD group revealed three significant differences. Non-Latinx caregivers were significantly more concerned about the pandemic's impact on childhood depression (*X^2 ^*= 6.67, *p < *.01), loneliness (*X^2 ^*= 6.89, *p < *.01), loss of social skills (*X^2 ^*= 7.72, *p < *.01), and too much screen time (*X^2 ^*= 4.30, *p < *.05). There was also a trend indicating that Latinx caregivers were possibly more concerned over catching COVID-19 (*X^2 ^*= 3.02, *p = *.08). DD-non-Latinx caregivers on average endorsed a greater number of pandemic-related concerns over DD-Latinx caregivers (*t^ ^*= −1.98, *p < *.05).

### Educational Concerns

 Table 3 provides information on educational concerns by demographic comparison groups. There were no significant group differences in educational concerns between parents of DD and TD children. Within the DD group, there was one marginally significant finding indicating non-Latinx caregivers of DD children may have been more concerned about their child missing out on social opportunities compared to Latinx caregivers of DD children (*X^2 ^*= 3.12, *p = *.08).

**Table 3.** Frequencies and chi-square results for educational concerns by demographic group.

Variable	DD	TD	X^2^	DD – Latinx	DD – Non-Latinx	X^2^
Child not ready to start K (% yes)	23	11	2.54	20	28	1.06
Child not gaining skills (% yes)	36	25	1.49	34	38	.15
Child may miss out on learning (% yes)	64	50	2.22	64	64	.00
Child may miss out on social opportunities (% yes)	78	81	.08	72	86	3.12^†^
Increase in challenging bxs (% yes)	59	50	.81	56	62	.44
Ability to transition back to school as usual (% yes)	58	56	.05	56	60	.20
Longer term health concerns for child (% yes)	17	8	1.65	21	12	1.68
Total Count (M:SD)	3.35(1.88)	2.81(1.77)	−1.53	3.23(1.88)	3.50(1.89)	−0.75

†*p* < .1 *p < .05 **p < .01 ***p < .001.

### Educational and Service Impact

 Table 4 shows the results from responses to the questions about COVID-19's impact on children's education and services. Results revealed five significant differences between the DD and TD groups. DD families were significantly more likely (*X^2 ^*= 7.01, *p < *.05) to spend more time involved in educational activities compared to TD families. For children who were enrolled in school and controlling for age, TD children spent significantly more time engaged in learning activities on a daily basis compared to DD children (*X^2 ^*= 14.11, *p < *.05). DD families also reported experiencing a greater decrease in early education services (*X^2 ^*= 24.53, *p < *.01) and a greater impact by the loss and/or delay of services on their child (*t* = −4.9, *p < *.01) compared to parents of TD children. DD caregivers also reported feeling significantly less capable to teach their child at home (*X^2 ^*= 9.33, *p < *.05), compared to TD caregivers. There was also a marginally significant difference in the involvement of alternative caregivers (e.g., older siblings, grandparents) were involved in the child's educational activities, with alternative caregivers in DD families being potentially more involved in the child's educational activities (*X^2 ^*= 6.54, *p = *.08) compared to TD families.

**Table 4.** Chi-Square results of COVID-19 educational impact questions.

Variable	DD/TD (X^2^)	DD – Latinx/Non-Latinx (X^2^)
How has your involvement changed	9.0^†^	5.20
Alternative caregiver's involvement in child's educational activities	6.54^†^	12.72*
How much time has your partner helped your children	1.35	4.29
How much time have you spent helping your children (under 4 h)	7.01*	15.23**
How much time have you spent helping your children (over 4 h)	.34	.05
How much time has your child engaged in school	14.11*	8.55*
How have your child's early education services changed	24.53**	1.53
Has the COVID-19 outbreak affected your regular childcare?	0.10	7.90
To what extent do you feel capable of teaching your child at home?	9.33*	11.15**
How much stress is remote instruction causing for your family?	2.20	0.32
How much has your child been impacted by a loss and/or delay of services on a scale 1–10 (*t*)	−4.9**	1.28

†*p* < .1 *p < .05 **p < .01 ***p < .001.

Within the DD sample, four variables revealed significant ethnic group differences. Controlling for caregiver employment status, Latinx caregivers spent significantly more time helping their child compared to non-Latinx families (*X^2 ^*= 15.23, *p < *.01). Also controlling for caregiver employment, non-Latinx caregivers were more likely to endorse that they felt “not at all” capable of doing at-home learning (*X^2 ^*= 11.15, *p = *.01) compared to Latinx caregivers. For children who were enrolled in school, non-Latinx families had greater involvement from an alternate caregiver (e.g., an older sibling, grandparent) in their child's educational activities (*X^2 ^*= 12.72, *p < *.05). Last, controlling for age, Latinx children spent more time engaged in school or learning activities compared to non-Latinx children (*X^2 ^*= 8.55, *p < *.05). Interestingly, reported stress levels from remote instruction were high across both groups of parents of children with DD, almost 50% of the total sample endorsing feeling “a lot” of stress from remote instruction compared to only 2% who felt “no” stress from remote instruction.

## Discussion

The current study aimed to describe general COVID-related and education specific concerns endorsed by a sample of diverse families with young children with and without DD. In addition, the study sought to determine if families of children with DD experienced more disruptions to education and services as compared to TD children, and whether ethnicity played a role in these lived experiences. We found that caregivers of children with DD endorsed a greater number of COVID and educational concerns compared to caregivers of TD children. This is an important finding as these additional COVID stressors may add to or exacerbate the broader challenges and high number of stressors that caregivers of children with DD typically experience. It is well documented in the literature that parenting stress is higher on average in caregivers of children with DD (Baker et al., 2002; Estes et al., 2009) and caregivers with higher levels of parenting stress pre-pandemic were more likely to report an increase in anxiety during the pandemic (Chan & Fung, 2022; Ren et al., 2020). Thus, there is likely a specific need for interventions that target with parenting stress and support the unique challenges that the pandemic has introduced. Future research should continue to explore challenges that caregivers are experiencing and the degree to which long-term pandemic-related parental concerns and anxieties about their children may be impacting caregiver mental health.

Non-Latinx caregivers of children with DD endorsed more COVID concerns relative to Latinx caregivers of children with DD, and non-Latinx caregivers also reported greater involvement from an alternate caregiver and endorsed feeling less capable of doing at-home learning. There are a number of possible factors that impacted caregiver responses to the stressors of navigating in-home learning. Latinx caregivers in the study were more likely to be stay-at-home caregivers (only 35% employed outside the home), and thus may be more accustomed to being involved in their child's educational activities. Although we controlled for caregiver employed outside of the home, familiarity of school-based routines (e.g., helping children with homework) may be influencing results. Other studies have found that caregivers of children with DD report feeling overwhelmed navigating at-home learning and serving as both caregiver and teaching (Valicenti-McDermott et al., 2022) and this effect may be magnified in caregivers who are not normally present in the home full-time or who previously relied on external support (e.g., childcare, other familial support).

Overall findings demonstrate that the transition to virtual learning and paucity of additional related services had a greater effect on caregivers of children with DD. These caregivers reported a greater impact from the loss and/or delay of services and reported feeling significantly less capable of conducting educational activities in the home. However, almost all caregivers in the study endorsed some level of stress from remote instruction and half of the total sample reported feeling “a lot” of stress. Thus, it is evident that families across the board were affected by the rapid transition to virtual learning and unprecedented challenges produced by the COVID-19 pandemic. These findings are corroborated by similar studies documenting challenges with remote learning practices for caregivers of children with DD during the initial stay-at-home order and school closures (Becker et al., 2020; Valicenti-McDermott et al., 2022). Now that schools have resumed in-person education, we may begin to see the long-term effects of the COVID-19 pandemic on educational and socio-emotional outcomes of children. There is a great need for accessible mental health services for families with young children and especially caregivers of children with DD (Ren et al., 2020). However, despite a great need, provisions for mental health services continue to fall short and there is absence of a coordinated plan for intervening on the ramifications of sustained levels of elevated anxiety (Peris & Ehrenreich-May, 2021).

## Limitations and Future Directions

There are a few limitations to note. First, results are derived from an online survey that was only distributed at one time point, early on in the pandemic and as such we are unable to comment on the longitudinal experience of families. In addition, although we controlled for demographic differences (e.g., child age), the sample had children in various stages of education and ranged from early childhood to school-aged. However, a strength of the study is the relatively high inclusion of Latinx and monolingual Spanish-speaking participants in the sample.

Future research should focus on elucidating the factors that have significantly contributed to parenting stress in order to tailor mental health interventions to the specific needs of caregivers of children with DD. Studies should also focus on the long-term impact of loss of services on children with DD and how this may affect school outcomes for high-risk children and families, including those with disabilities.

Caregivers of young children experienced a number of challenges and stressors by the transition to virtual learning during the COVID-19 pandemic stay-at-home order. Moreover, parents of children with DD faced additional challenges, as these children are more likely to require specialized materials and accessed special education and services, most of which were halted or significantly reduced. Thus, it was important to understand the challenges that caregivers of young children experienced throughout the pandemic and school closures. Our findings provide important information for educators and service professionals to respond to the present challenges experienced by caregivers.
